# The Story of the Insane from Year to Year

**Published:** 1896-01-18

**Authors:** 


### THE STORY OF THE INSANE FROM YEAR TO YEAR.

### EIGHTH SERIES.

### ABERDEEN ROYAL ASYLUM.

At the beginning of the year this asylum contained 686 patients, and by the end of the year the number had risen to 714. There were 208 admissions, 83 recoveries, 17 relieved, and 49 deaths. These numbers show that 39·9 per cent. of the admissions recovered, and that 7 per cent. of the average number resident died. Of the deaths 19 were caused by cerebral and spinal diseases, and 5 by consumption. Intemperance was the cause of the insanity in 18 of the admissions, and hereditary influence in 62. Dr. Reid truly says that if the causes could be correctly estimated hereditary influence would play a still more important part, and he adds that "a study of the causes of insanity leads us to the conclusion that the chief agency is nerve instability transmitted from parent to progeny. The greater the force of this instability the less need be the immediately exciting causes which produce disorder of brain function." The whole passage on this subject is extremely well put, and we regret being unable to quote more of it. Lectures and instructions are given to the attendants and nurses, and four of the former and six of the latter gained the Medico-Psychological certificate. Dr. Reid seems to think the requirement as to length of service by the association is too long. We think it is too short, and in no case should anyone be allowed to possess the certificate under three years' service in the same asylum.

NORTHAMPTON COUNTY ASYLUM.

Eight hundred and fifty-nine patients were in residence on January 1st, and by the end of the year the number had fallen to 836, owing to the removal of some out-county cases. There were 242 admissions, 51 recoveries, and 71 deaths. The recoveries stand at 29 per cent. on the admissions, and the deaths at 8·6 per cent. on the average number resident. Of the deaths 37 were caused by cerebral or spinal diseases, and 6 by consumption. Domestic trouble and religion account for 11 of the year's admissions, hereditary influence for 76, and drink for 12. We learn that the children's block at this asylum is now quite full, and that "50 per cent. of the children have been greatly improved in their habits; about the same proportion attend the schools regularly, and four have been discharged recovered, taking with them from the asylum sufficient knowledge of laundry or domestic work to be a great help to their parents." This is an encouraging statement to those who have built or are about to build for idiot children. We also learn that thirteen nurses have obtained their nursing certificates. These nurses obtain higher pay than the others, and Mr. Greene says that this "acknowledgment of the increased value of their services has stimulated others to theoretical and practical study. Five nurses are now in their third year of training, ten in their second year, and ten their first." The weekly cost of maintenance is 7s. 9½d. per head.

DERBY BOROUGH ASYLUM.

Here there was only a slight increase in the numbers. On January 1st 306 patients were in residence, and at the end of the year 310. There were 86 admissions, 41 recoveries, 8 relieved, and 27 deaths. The recoveries reach the high percentage of 47·3, calculated on the admissions, and the deaths stand at 11·4 on the average number resident. Fourteen of the deaths were caused by cerebral and spinal diseases, 4 by inflammation of the lungs, and only 1 was due to consumption. Of those admitted during the year 26 were driven mad by drink, and 22 by hereditary influence, while domestic troubles and mental anxiety are credited with being the cause in 13. The asylum is full on the women's side and almost so on the men's, but 94 of the patients do not belong to the borough of Derby. The committee say they have "much pleasure in renewing the expression of their entire satisfaction with the manner in which Dr. Macphail continues to discharge the responsible and onerous duties which devolve upon him." The Commissioners' report is not given. The weekly cost per head is 9s. 6d.

